# The Fractional Differential Model of HIV-1 Infection of CD4^+^ T-Cells with Description of the Effect of Antiviral Drug Treatment

**DOI:** 10.1155/2019/4059549

**Published:** 2019-01-08

**Authors:** Bijan Hasani Lichae, Jafar Biazar, Zainab Ayati

**Affiliations:** ^1^Department of Mathematics, Guilan Science and Research Branch, Islamic Azad University, Rasht, Iran; ^2^Department of Mathematics, Rasht Branch, Islamic Azad University, Rasht, Iran; ^3^Department of Applied Mathematics, Faculty of Mathematical Sciences, University of Guilan, P.O. Box 41335-1914, Rasht, Guilan, Iran; ^4^Department of Engineering Sciences, Faculty of Technology and Engineering East of Guilan, University of Guilan, P.C. 44891-63157, Rudsar-Vajargah, Iran

## Abstract

In this paper, the fractional-order differential model of HIV-1 infection of CD4^+^ T-cells with the effect of drug therapy has been introduced. There are three components: uninfected CD4^+^ T-cells, *x*, infected CD4^+^ T-cells, *y*, and density of virions in plasma, *z*. The aim is to gain numerical solution of this fractional-order HIV-1 model by Laplace Adomian decomposition method (LADM). The solution of the proposed model has been achieved in a series form. Moreover, to illustrate the ability and efficiency of the proposed approach, the solution will be compared with the solutions of some other numerical methods. The Caputo sense has been used for fractional derivatives.

## 1. Introduction

Human immunodeficiency virus (HIV) is a retrovirus that causes acquired immunodeficiency syndrome (AIDS) [1]. HIV infects, damages, and reduces CD4^+^ T-cells. Therefore, it causes to decrease the resistance of immune system [2]. The body becomes more gradually sensitive to infections and loses its safety. AIDS is one the most important and dangerous diseases in our time. According to UNAIDS 2017 annual report, “36.7 million people globally were living with HIV and 1.8 million people became newly infected with HIV and 1 million people died from AIDS-related illnesses in 2016.” In spite of the great progress in controlling the disease, no vaccine has been yet discovered for HIV. In the last two decades, a lot of efforts have been made to design and solve mathematical models that have essential rule in analyzing to control and prevent the spread of HIV-related diseases [3–13]. Usually almost all of these mathematical models explain the relation between HIV viruses and uninfected CD4^+^ cells and the effect of drug therapy to infected cells. Bonhoeffer et al. [4] presented a model for virus dynamics with two components *x* and *y*, where *x* denotes the density of infected cells and *y* shows the density of virus-producing cells.

The proposed model is as follows:

(1) $$\begin{array}{c} \left\{\begin{array}{c} \frac{dx}{dt}=c-\beta x-\gamma xy,\\ \frac{dy}{dt}=\gamma xy-dy, \end{array}\right. \end{array}$$

where *c* is the rate of production of infected cells, *β* is the natural death rate of infected cells, *d* is the rate of virus-producing cells' death, and *γ* is the rate of infection of uninfected cells. This model and many such models were inspired from Anderson's model [14, 15]. Anderson's model is one of the first and the most important models of infectious diseases. Tuckwell and Wan [16] introduced a modified model of equation (1) with three components: uninfected, infected CD4^+^ T-cells, and density of virions in plasma (*x*, *y*, and *z*, respectively). The presented model with three equations is as follows:

(2) $$\begin{array}{c} \left\{\begin{array}{c} \frac{dx}{dt}=s^{\prime }-\mu x-\beta xz,\\ \frac{dy}{dt}=\beta xz-\varepsilon y,\\ \frac{dz}{dt}=cy-\gamma z, \end{array}\right. \end{array}$$

with the initial conditions *x*(0)=*k*_1_,*y*(0)=*k*_2_, and *z*(0)=*k*_3_ where *s*′, *μ*, *β*, *ε*, *c*, and *γ* are constant coefficients, *s*′ is the rate of creation or production of CD4^+^ T-cells, *μ* is the natural death rate, *β* is the rate of infected CD4^+^ cells from uninfected CD4^+^ cells, *ε* is the rate of virus-producing cells' death, *c* is the rate of creation of virions viruses by infected cells, and *γ* is the rate of virus particle death. For the sake of comparison and showing the ability of the proposed approach, we use the parameter values reported in references [6, 16]. The parameter values are as follows: *s*′=0.272 (day/mm^3^), *μ*=0.00136 (day/mm^3^), *β*=0.00027 (day/virion/mm^3^), *ε*=0.33 (day/mm^3^), *c*=50 (virion/CLM/day), and *γ*=2.0 (day). The rate of some coefficients will change if drug therapy is not 100% successful. When the drug treatment begins, infected cells which create virus components are affected. If the drug therapy is not effective, a part of infected cells will improve and remaining cells will begin to produce virus [8].

Mathematical modeling of many problems in biology and other branches of sciences appears as differential equations in fractional order. Because the fractional-order differential equations save memory on themselves and are related to fractals [8, 17–19], we prefer to use the fractional-order form of the model (2) as follows:

(3) $$\begin{array}{c} \left\{\begin{array}{c} D^{\alpha_{1}}\left(x\right)=s^{\prime }-\mu x-\beta xz,\\ D^{\alpha_{2}}\left(y\right)=\beta xz-\varepsilon y,\\ D^{\alpha_{3}}\left(z\right)=cy-\gamma z, \end{array}\right. \end{array}$$

with the same initial conditions, where 0 < *α*_*i*_ ≤ 1, *i*=1,2,3. There are some numerical approaches for solving these types of mathematical models. Some of these methods are as follows: homotopy analysis, optimal homotopy asymptotic, homotopy perturbation, Adomian decomposition, and variational iteration [20–30]. In [8], system of fractional equation (3) has been solved by homotopy analysis method (HAM) and generalized Euler method (GEM). In [31], equation (3) has also been solved by homotopy perturbation method (HPM). Adomian [32], introduced a decomposition method (ADM) which is a powerful method to get analytic approximate solution of differential equations. Using Laplace transform method with couple of ADM (LADM) to solve systems of differential equations leads to an effective method that finds many applications in applied mathematics. In this paper, we will solve equation (3) by LADM and will compare the results with the results achieved by generalized Euler, homotopy analysis, homotopy perturbation, and Runge–Kutta methods. The structure of the paper is as follows: in Section 2, we will present a brief review of fractional calculus. In Sections 3 and 4, we will solve the fractional-order HIV-1 model by LADM. In Section 5, the convergence of the method will be discussed. In the last section, we present the conclusion.

## 2. Fractional Calculus

The purpose of this section is to recall a few preliminaries about what appears in this research.

**Definition 1 .**

The Riemann–Liouville fractional integral of order *α* for a function *X* : (0, *∞*)⟶*R* is defined as

(4) $$\begin{array}{c} J^{\alpha }X\left(s\right)=\frac{1}{\Gamma \left(\alpha \right)}\int_{0}^{s}{\left(s-t\right)}^{\alpha -1}X\left(t\right)dt, \end{array}$$

where *αε*(0, *∞*) (see [33]).

**Definition 2 .**

The Caputo fractional derivative for a function *X* : (0, *∞*)⟶*R* on the closed interval [0, *S*] is defined as

(5) $$\begin{array}{c} D^{\alpha }X\left(s\right)=\frac{1}{\Gamma \left(m-\alpha \right)}\int_{0}^{s}{\left(s-t\right)}^{m-\alpha -1}X^{m}\left(t\right)dt,\;m=\left\lfloor \alpha \right\rfloor +1, \end{array}$$

where *α* is the integer part of *α*. Another presentation of the Caputo fractional derivative can be shown as follows (see [33]):

(6) $$\begin{array}{c} D^{\alpha }X\left(s\right)=J^{m-\alpha }\left(D^{m}X\left(s\right)\right). \end{array}$$

**Lemma 1 .**

If *αε*(0, *∞*) and *m*=*α*+1, then the following result holds for fractional calculus:

(7) $$\begin{array}{c} J^{\alpha }\left[D^{\alpha }X\right]\left(s\right)=X\left(s\right)+\overset{m-1}{\underset{j=0}{\sum }}\frac{X^{j}\left(0\right)}{j!}s^{j}. \end{array}$$

**Proof**

See [33, 34].

**Definition 3 .**

The Laplace transform of Caputo fractional derivative is defined as follows:

(8) $$\begin{array}{c} ℒ\left\{D^{\alpha }X\left(t\right)\right\}=s^{\alpha }Y\left(s\right)-\overset{m-1}{\underset{j=0}{\sum }}s^{\alpha -k-1}X^{j}\left(0\right),\;m-1<\alpha <m,m\in \mathbb{N}. \end{array}$$

## 3. Solution of Model (3)

In this section, LADM has been implemented to solve system of fractional equation (3) with the initial conditions.

We apply Laplace transform on both sides of each equation of equation (3):

(9) $$\begin{array}{c} \left\{\begin{array}{c} L\left\{D^{\alpha_{1}}x\right\}=L\left\{s^{\prime }-\mu x-\beta xz\right\},\\ L\left\{D^{\alpha_{2}}y\right\}=L\left\{\beta xz-\varepsilon y\right\},\\ L\left\{D^{\alpha_{3}}z\right\}=L\left\{cy-\gamma z\right\}, \end{array}\right. \end{array}$$

which implies that

(10) $$\begin{array}{c} \left\{\begin{array}{c} s^{\alpha_{1}}L\left\{x\right\}-s^{\alpha_{1}-1}x\left(0\right)=L\left\{s^{\prime }-\mu x-\beta xz\right\},\\ s^{\alpha_{2}}L\left\{y\right\}-s^{\alpha_{2}-1}y\left(0\right)=L\left\{\beta xz-\varepsilon y\right\},\\ s^{\alpha_{3}}L\left\{z\right\}-s^{\alpha_{3}-1}z\left(0\right)=L\left\{cy-\gamma z\right\}. \end{array}\right. \end{array}$$

Substitution of the initial conditions in equation (10) and applying inverse Laplace transform results in

(11) $$\begin{array}{c} \left\{\begin{array}{c} x=k_{1}+{ℒ}^{-1}\left[\frac{1}{s^{\alpha_{1}}}ℒ\left\{s^{\prime }-\mu x-\beta xz\right\}\right],\\ y=k_{2}+{ℒ}^{-1}\left[\frac{1}{s^{\alpha_{2}}}ℒ\left\{\beta xz-\varepsilon y\right\}\right],\\ z=k_{3}+{ℒ}^{-1}\left[\frac{1}{s^{\alpha_{3}}}ℒ\left\{cy-\gamma z\right\}\right]. \end{array}\right. \end{array}$$

To apply ADM, let us consider *x*, *y*, and *z* as the following series:

(12) $$\begin{array}{c} x=\overset{\infty }{\underset{i=0}{\sum }}x_{i},\\ y=\overset{\infty }{\underset{i=0}{\sum }}y_{i},\\ z=\overset{\infty }{\underset{i=0}{\sum }}z_{i}. \end{array}$$

To decompose the nonlinear term *xz*, let us follow an alternate algorithm [35] to get,

(13) $$\begin{array}{c} xz=\overset{\infty }{\underset{i=0}{\sum }}p_{i}, \end{array}$$

where *p*_*i*_ is as the following equation:

(14) $$\begin{array}{c} p_{i}=\overset{i}{\underset{k=0}{\sum }}x_{k}z_{i-k}, \end{array}$$

substituting equations (12)–(14) into (11) reads

(15) $$\begin{array}{c} \left\{\begin{array}{c} ℒ\left(x_{0}\right)=\frac{k_{1}}{s},\\ ℒ\left(y_{0}\right)=\frac{k_{2}}{s},\\ ℒ\left(z_{0}\right)=\frac{k_{3}}{s},\\ ℒ\left(x_{1}\right)=\frac{s^{\prime }}{s^{\alpha_{1}+1}}-\frac{\mu }{s^{\alpha_{1}}}ℒ\left(x_{0}\right)-\frac{\beta }{s^{\alpha_{1}}}ℒ\left(p_{0}\right),\\ ℒ\left(y_{1}\right)=\frac{\beta }{s^{\alpha_{2}}}ℒ\left(p_{0}\right)-\frac{\varepsilon }{s^{\alpha_{2}}}ℒ\left(y_{0}\right),\\ ℒ\left(z_{1}\right)=\frac{c}{s^{\alpha_{3}}}ℒ\left(y_{0}\right)-\frac{\gamma }{s^{\alpha_{3}}}ℒ\left(z_{0}\right),\\ ℒ\left(x_{2}\right)=-\frac{\mu }{s^{\alpha_{1}}}ℒ\left(x_{1}\right)-\frac{\beta }{s^{\alpha_{1}}}ℒ\left(p_{1}\right),\\ ℒ\left(y_{2}\right)=\frac{\beta }{s^{\alpha_{2}}}ℒ\left(p_{1}\right)-\frac{\varepsilon }{s^{\alpha_{2}}}ℒ\left(y_{1}\right),\\ ℒ\left(z_{2}\right)=\frac{c}{s^{\alpha_{3}}}ℒ\left(y_{1}\right)-\frac{\gamma }{s^{\alpha_{3}}}ℒ\left(z_{1}\right),\\ \vdots \\ ℒ\left(x_{n+1}\right)=-\frac{\mu }{s^{\alpha_{1}}}ℒ\left(x_{n}\right)-\frac{\beta }{s^{\alpha_{1}}}ℒ\left(p_{n}\right),\\ ℒ\left(y_{n+1}\right)=\frac{\beta }{s^{\alpha_{2}}}ℒ\left(p_{n}\right)-\frac{\varepsilon }{s^{\alpha_{2}}}ℒ\left(y_{n}\right),\\ ℒ\left(z_{n+2}\right)=\frac{c}{s^{\alpha_{3}}}ℒ\left(y_{n}\right)-\frac{\gamma }{s^{\alpha_{3}}}ℒ\left(z_{n}\right). \end{array}\right. \end{array}$$

We take inverse Laplace transform on both sides of each equation of equation (15):

(16) $$\begin{array}{c} x_{0}=k_{1},\\ y_{0}=k_{2},\\ z_{0}=k_{3},\\ x_{1}=\left(s^{\prime }-\mu x_{0}-\beta x_{0}z_{0}\right)\frac{t^{\alpha_{1}}}{\Gamma \left(\alpha_{1}+1\right)},\\ y_{1}=\left(\beta x_{0}z_{0}-\varepsilon y_{0}\right)\frac{t^{\alpha_{2}}}{\Gamma \left(\alpha_{2}+1\right)},\\ z_{1}=\left(cy_{0}-\gamma z_{0}\right)\frac{t^{\alpha_{3}}}{\Gamma \left(\alpha_{3}+1\right)},\\ x_{2}=\left\{-\left(\mu +\beta z_{0}\right)\left(s^{\prime }-\mu x_{0}-\beta x_{0}z_{0}\right)\right\}\frac{t^{2\alpha_{2}}}{\Gamma \left(2\alpha_{2}+1\right)}-\beta x_{0}\left(cy_{0}-\gamma z_{0}\right)\frac{t^{\alpha_{1}+\alpha_{3}}}{\Gamma \left(\alpha_{1}+\alpha_{3}+1\right)},\\ y_{2}=\beta x_{0}\left(cy_{0}-\gamma z_{0}\right)\frac{t^{\alpha_{2}+\alpha_{3}}}{\Gamma \left(\alpha_{2}+\alpha_{3}+1\right)}+\beta z_{0}\left(s^{\prime }-\mu x_{0}-\beta x_{0}z_{0}\right)\frac{t^{\alpha_{1}+\alpha_{2}}}{\Gamma \left(\alpha_{1}+\alpha_{2}+1\right)}-\varepsilon \left(\beta x_{0}z_{0}-\varepsilon y_{0}\right)\frac{t^{2\alpha_{2}}}{\Gamma \left(2\alpha_{2}+1\right)},\\ z_{2}=c\left(\beta x_{0}z_{0}-\varepsilon y_{0}\right)\frac{t^{\alpha_{2}+\alpha_{3}}}{\Gamma \left(\alpha_{2}+\alpha_{3}+1\right)}-\gamma \left(cy_{0}-\gamma z_{0}\right)\frac{t^{2\alpha_{3}}}{\Gamma \left(2\alpha_{3}+1\right)},\\ x_{3}=\beta x_{0}c\left(\beta x_{0}z_{0}-\varepsilon y_{0}\right)\frac{t^{\alpha_{1}+\alpha_{2}+\alpha_{3}}}{\Gamma \left(\alpha_{1}+\alpha_{2}+\alpha_{3}+1\right)}+\beta x_{0}\gamma \left(cy_{0}-\gamma z_{0}\right)\frac{t^{\alpha_{1}+2\alpha_{3}}}{\Gamma \left(\alpha_{1}+2\alpha_{3}+1\right)}+\beta^{2}x_{0}z_{0}\left(cy_{0}-\gamma z_{0}\right)\frac{t^{2\alpha_{1}+\alpha_{2}}}{\Gamma \left(2\alpha_{1}+\alpha_{2}+1\right)}+\left(s^{\prime }-\mu x_{0}-\beta x_{0}z_{0}\right)\left\{\beta z_{0}\left(\mu +z_{0}\right)+\mu \left(\mu +\beta z_{0}\right)\right\}\frac{t^{3\alpha_{1}}}{\Gamma \left(3\alpha_{1}+1\right)}+\beta \left\{\left(cy_{0}-\gamma z_{0}\right)\left(\mu x_{0}-\left(s^{\prime }-\mu x_{0}-\beta x_{0}z_{0}\right)\frac{\Gamma \left(\alpha_{1}+\alpha_{3}+1\right)}{\Gamma \left(\alpha_{1}+1\right)\Gamma \left(\alpha_{3}+1\right)}\right)\right\}\frac{t^{2\alpha_{1}+\alpha_{3}}}{\Gamma \left(2\alpha_{1}+\alpha_{3}+1\right)},\\ y_{3}=\left(\beta x_{0}\left(c\left(\beta x_{0}z_{0}-\varepsilon y_{0}\right)-\varepsilon \left(cy_{0}-\gamma z_{0}\right)\right)\right)\frac{t^{2\alpha_{2}+\alpha_{3}}}{\Gamma \left(2\alpha_{2}+\alpha_{3}+1\right)}-\beta x_{0}\gamma \left(cy_{0}-\gamma z_{0}\right)\frac{t^{\alpha_{2}+2\alpha_{3}}}{\Gamma \left(\alpha_{2}+2\alpha_{3}+1\right)}-\beta z_{0}\left(\mu +\beta z_{0}\right)\left(s^{\prime }-\mu x_{0}-\beta x_{0}z_{0}\right)\frac{t^{2\alpha_{1}+\alpha_{2}}}{\Gamma \left(2\alpha_{1}+\alpha_{2}+1\right)}+\beta \left(s^{\prime }-\mu x_{0}-\beta x_{0}z_{0}\right)\frac{\Gamma \left(\alpha_{1}+\alpha_{3}+1\right)}{\Gamma \left(\alpha_{1}+1\right)\Gamma \left(\alpha_{3}+1\right)}\frac{t^{\alpha_{1}+\alpha_{2}+\alpha_{3}}}{\Gamma \left(\alpha_{1}+\alpha_{2}+\alpha_{3}+1\right)}-\left(\beta^{2}x_{0}z_{0}\left(cy_{0}-\gamma z_{0}\right)+\beta z_{0}\varepsilon \left(s^{\prime }-\mu x_{0}-\beta x_{0}z_{0}\right)\right)\frac{t^{\alpha_{1}+2\alpha_{2}}}{\Gamma \left(\alpha_{1}+2\alpha_{2}+1\right)}+\varepsilon^{2}\left(\beta x_{0}z_{0}-\varepsilon y_{0}\right)\frac{t^{3\alpha_{2}}}{\Gamma \left(3\alpha_{2}+1\right)},\\ z_{3}=\left(\beta x_{0}c\left(cy_{0}-\gamma z_{0}\right)-\gamma c\left(\beta x_{0}z_{0}-\varepsilon y_{0}\right)\right)\frac{t^{\alpha_{2}+2\alpha_{3}}}{\Gamma \left(\alpha_{2}+2\alpha_{3}+1\right)}+\gamma^{2}\left(cy_{0}-\gamma z_{0}\right)\frac{t^{3\alpha_{3}}}{\Gamma \left(3\alpha_{3}+1\right)}+\beta z_{0}c\left(s^{\prime }-\mu x_{0}-\beta x_{0}z_{0}\right)\frac{t^{\alpha_{1}+\alpha_{2}+\alpha_{3}}}{\Gamma \left(\alpha_{1}+\alpha_{2}+\alpha_{3}+1\right)}-\varepsilon c\left(\beta x_{0}z_{0}-\varepsilon y_{0}\right)\frac{t^{2\alpha_{2}+\alpha_{3}}}{\Gamma \left(2\alpha_{2}+\alpha_{3}+1\right)}. \end{array}$$

We have calculated four terms of the infinite series of *x*, *y*, and *z* as an approximate solution. To get any desired accuracy, one is able to proceed the process and obtain more terms. Finally, the solution of mathematical model can be obtained as follows:

(17) $$\begin{array}{c} \left\{\begin{array}{c} x\left(t\right)=\overset{\infty }{\underset{i=0}{\sum }}x_{i}\left(t\right)\approx x_{0}\left(t\right)+x_{1}\left(t\right)+x_{2}\left(t\right)+x_{3}\left(t\right),\\ y\left(t\right)=\overset{\infty }{\underset{i=0}{\sum }}y_{i}\left(t\right)\approx y_{0}\left(t\right)+y_{1}\left(t\right)+y_{2}\left(t\right)+y_{3}\left(t\right),\\ z\left(t\right)=\overset{\infty }{\underset{i=0}{\sum }}z_{i}\left(t\right)\approx z_{0}\left(t\right)+z_{1}\left(t\right)+z_{2}\left(t\right)+z_{3}\left(t\right). \end{array}\right. \end{array}$$

## 4. Numerical Simulation

In this section, constants and initial values are substituted in equation (16) to obtain an approximate solution.

Substituting the following values: *s*′  = 0.272 (day/mm^3^), *μ* = 0.00136 (day/mm^3^), *β* = 0.00027 (day/virion/mm^3^), *ε* = 0.33 (day/mm^3^), *c* = 50 (virion/CLM/day), and *γ* = 2.0 (day) and the initial conditions *x*(0)=100, *y*(0)=0, and *z*(0)=1 in equation (16), we get,

(18) $$\begin{array}{c} x_{0}=100,\\ y_{0}=0,\\ z_{0}=1,\\ x_{1}=0.1090\frac{t^{\alpha_{1}}}{\Gamma \left(\alpha_{1}+1\right)},\\ y_{1}=0.0270\frac{t^{\alpha_{2}}}{\Gamma \left(\alpha_{2}+1\right)},\\ z_{1}=-2.0000\frac{t^{\alpha_{3}}}{\Gamma \left(\alpha_{3}+1\right)},\\ x_{2}=-0.0001776700\frac{t^{2\alpha 2}}{\Gamma \left(2\alpha_{2}+1\right)}+0.05400\frac{t^{\alpha_{1}+\alpha_{3}}}{\Gamma \left(\alpha_{1}+\alpha_{3}+1\right)},\\ y_{2}=-0.05400\frac{t^{\alpha_{2}+\alpha_{3}}}{\Gamma \left(\alpha_{2}+\alpha_{3}+1\right)}+0.0000294300\frac{t^{\alpha_{1}+\alpha_{2}}}{\Gamma \left(\alpha_{1}+\alpha_{2}+1\right)}-0.0089100\frac{t^{2\alpha_{2}}}{\Gamma \left(2\alpha_{2}+1\right)},\\ z_{2}=1.35000\frac{t^{\alpha_{2}+\alpha_{3}}}{\Gamma \left(\alpha_{2}+\alpha_{3}+1\right)}+4.0000\frac{t^{2\alpha_{3}}}{\Gamma \left(2\alpha_{3}+1\right)},\\ x_{3}=-0.0364500\frac{t^{\alpha_{1}+\alpha_{2}+\alpha_{3}}}{\Gamma \left(\alpha_{1}+\alpha_{2}+\alpha_{3}+1\right)}-0.10800\frac{t^{\alpha_{1}+2\alpha_{3}}}{\Gamma \left(\alpha_{1}+2\alpha_{3}+1\right)}-0.0000145800\frac{t^{2\alpha_{1}+2\alpha_{2}}}{\Gamma \left(2\alpha_{1}+\alpha_{2}+1\right)}+0.000029711656\frac{t^{3\alpha_{1}}}{\Gamma \left(3\alpha_{1}+1\right)}-0.00054\frac{\Gamma \left(\alpha_{1}+\alpha_{3}+1\right)}{\Gamma \left(\alpha_{1}+1\right)\Gamma \left(\alpha_{3}+1\right)}\frac{t^{2\alpha_{1}+\alpha_{3}}}{\Gamma \left(2\alpha_{1}+\alpha_{3}+1\right)},\\ y_{3}=0.054270000\frac{t^{2\alpha_{2}+\alpha_{3}}}{\Gamma \left(2\alpha_{2}+\alpha_{3}+1\right)}+0.10800\frac{t^{\alpha_{2}+2\alpha_{3}}}{\Gamma \left(\alpha_{2}+2\alpha_{3}+1\right)}-0.000000047971\frac{t^{2\alpha_{1}+\alpha_{2}}}{\Gamma \left(2\alpha_{1}+\alpha_{2}+1\right)}+0.0000294300\frac{\Gamma \left(\alpha_{1}+\alpha_{3}+1\right)}{\Gamma \left(\alpha_{1}+1\right)\Gamma \left(\alpha_{3}+1\right)}\frac{t^{\alpha_{1}+\alpha_{2}+\alpha_{3}}}{\Gamma \left(\alpha_{1}+\alpha_{2}+\alpha_{3}+1\right)}+\text{0}.000004868100\frac{t^{\alpha_{1}+2\alpha_{3}}}{\Gamma \left(\alpha_{1}+2\alpha_{3}+1\right)}+\text{0}.0002940300\frac{t^{3\alpha_{2}}}{\Gamma \left(3\alpha_{2}+1\right)},\\ z_{3}=\text{0}.054270000\frac{t^{\alpha_{2}+2\alpha_{3}}}{\Gamma \left(\alpha_{2}+2\alpha_{3}+1\right)}-8.0000\frac{t^{3\alpha_{3}}}{\Gamma \left(3\alpha_{3}+1\right)}+\text{0}.0014715000\frac{t^{\alpha_{1}+\alpha_{2}+\alpha_{3}}}{\Gamma \left(\alpha_{1}+\alpha_{2}+\alpha_{3}+1\right)}-\text{0}.4455000\frac{t^{2\alpha_{2}+\alpha_{3}}}{\Gamma \left(2\alpha_{2}+\alpha_{3}+1\right)}. \end{array}$$

Three terms approximations can be written as the following form:

(19) $$\begin{array}{c} x\left(t\right)=100+0.1090\frac{t^{\alpha_{1}}}{\Gamma \left(\alpha_{1}+1\right)}-\text{0}.0001776700\frac{t^{{2\alpha }_{2}}}{\Gamma \left({2\alpha }_{2}+1\right)}+\text{0}.05400\frac{t^{\alpha_{1}+\alpha_{3}}}{\Gamma \left(\alpha_{1}+\alpha_{3}+1\right)}-\text{0}.0364500\frac{t^{\alpha_{1}+\alpha_{2}+\alpha_{3}}}{\Gamma \left(\alpha_{1}+\alpha_{2}+\alpha_{3}+1\right)}-\text{0}.10800\frac{t^{\alpha_{1}+2\alpha_{3}}}{\Gamma \left(\alpha_{1}+2\alpha_{3}+1\right)}-\text{0}.0000145800\frac{t^{2\alpha_{1}+\alpha_{2}}}{\Gamma \left(2\alpha_{1}+\alpha_{2}+1\right)}+\text{0}.000029711656\frac{t^{3\alpha_{1}}}{\Gamma \left(3\alpha_{1}+1\right)}-\text{0}.00054\frac{\Gamma \left(\alpha_{1}+\alpha_{3}+1\right)}{\Gamma \left(\alpha_{1}+1\right)\Gamma \left(\alpha_{3}+1\right)}\frac{t^{2\alpha_{1}+\alpha_{3}}}{\Gamma \left(2\alpha_{1}+\alpha_{3}+1\right)},\\ y\left(t\right)=\text{0}.0270\frac{t^{\alpha_{2}}}{\Gamma \left(\alpha_{2}+1\right)}-\text{0}.05400\frac{t^{\alpha_{2}+\alpha_{3}}}{\Gamma \left(\alpha_{2}+\alpha_{3}+1\right)}+\text{0}.0000294300\frac{t^{\alpha_{1}+\alpha_{2}}}{\Gamma \left(\alpha_{1}+\alpha_{2}+1\right)}-\text{0}.0089100\frac{t^{2\alpha_{2}}}{\Gamma \left(2\alpha_{2}+1\right)}+\text{0}.054270000\frac{t^{2\alpha_{2}+\alpha_{3}}}{\Gamma \left(2\alpha_{2}+\alpha_{3}+1\right)}+\text{0}.10800\frac{t^{\alpha_{2}+2\alpha_{3}}}{\Gamma \left(\alpha_{2}+2\alpha_{3}+1\right)}-\text{0}.000000047971\frac{t^{2\alpha_{1}+\alpha_{2}}}{\Gamma \left(2\alpha_{1}+\alpha_{2}+1\right)}+\text{0}.0000294300\frac{\Gamma \left(\alpha_{1}+\alpha_{3}+1\right)}{\Gamma \left(\alpha_{1}+1\right)\Gamma \left(\alpha_{3}+1\right)}\frac{t^{\alpha_{1}+\alpha_{2}+\alpha_{3}}}{\Gamma \left(\alpha_{1}+\alpha_{2}+\alpha_{3}+1\right)}+\text{0}.000004868100\frac{t^{\alpha_{1}+2\alpha_{2}}}{\Gamma \left(\alpha_{1}+2\alpha_{2}+1\right)}+\text{0}.0002940300\frac{t^{3\alpha_{2}}}{\Gamma \left(3\alpha_{2}+1\right)},\\ z\left(t\right)=1-2.0000\frac{t^{\alpha_{3}}}{\Gamma \left(\alpha_{3}+1\right)}+1.35000\frac{t^{\alpha_{2}+\alpha_{3}}}{\Gamma \left(\alpha_{2}+\alpha_{3}+1\right)}+4.0000\frac{t^{2\alpha_{3}}}{\Gamma \left(2\alpha_{3}+1\right)}+\text{0}.054270000\frac{t^{\alpha_{2}+2\alpha_{3}}}{\Gamma \left(\alpha_{2}+2\alpha_{3}+1\right)}-8.0000\frac{t^{3\alpha_{3}}}{\Gamma \left(3\alpha_{3}+1\right)}+\text{0}.0014715000\frac{t^{\alpha_{1}+\alpha_{2}+\alpha_{3}}}{\Gamma \left(\alpha_{1}+\alpha_{2}+\alpha_{3}+1\right)}-\text{0}.4455000\frac{t^{2\alpha_{2}+\alpha_{3}}}{\Gamma \left(2\alpha_{2}+\alpha_{3}+1\right)}. \end{array}$$

Let us take *α*_1_, *α*_2_, and *α*_3_ equal to *α*, so the solution of fractional-order of model (3) is obtained as follows:

(20) $$\begin{array}{c} \left\{\begin{array}{c} x\left(t\right)=100+\frac{0.1090t^{\alpha }}{\Gamma \left(\alpha +1\right)}+\frac{0.0538223300t^{2\alpha }}{\Gamma \left(2\alpha +1\right)}-\frac{0.1444348683t^{3\alpha }}{\Gamma \left(3\alpha +1\right)}-\frac{0.00054\Gamma \left(2\alpha +1\right)t^{3\alpha }}{\Gamma \left(3\alpha +1\right)},\\ y\left(t\right)=\frac{0.0270t^{\alpha }}{\Gamma \left(\alpha +1\right)}-\frac{0.0628805700t^{2\alpha }}{\Gamma \left(2\alpha +1\right)}+\frac{0.1625688501t^{3\alpha }}{\Gamma \left(3\alpha +1\right)}+\frac{0.0000294300\Gamma \left(2\alpha +1\right)t^{3\alpha }}{\Gamma \left(3\alpha +1\right)},\\ z\left(t\right)=1-\frac{2.0000t^{\alpha }}{\Gamma \left(\alpha +1\right)}+\frac{5.35000t^{2\alpha }}{\Gamma \left(2\alpha +1\right)}-\frac{8.389758500t^{3\alpha }}{\Gamma \left(3\alpha +1\right)}. \end{array}\right. \end{array}$$

For *α*_1_=*α*_2_=*α*_3_=1, the solution of equation (3) will be as follows:

(21) $$\begin{array}{c} \left\{\begin{array}{c} x\left(t\right)=100+0.1090t+0.02691116500t^{2}-0.02425247806t^{3},\\ y\left(t\right)=0.0270t-0.03144028500t^{2}+0.02710461836t^{3},\\ z\left(t\right)=1-2.0000t+2.6750000t^{2}-1.398293083t^{3}. \end{array}\right. \end{array}$$

In Tables 1–3, one can compare the approximate solution of fractional-order of model (3) with the results of GEM, HAM, RK4 in [8], and HPM in [31] using traditional order *α*=1. The results of LADM are more accurate than the results obtained by other methods.

**Figure 1:**
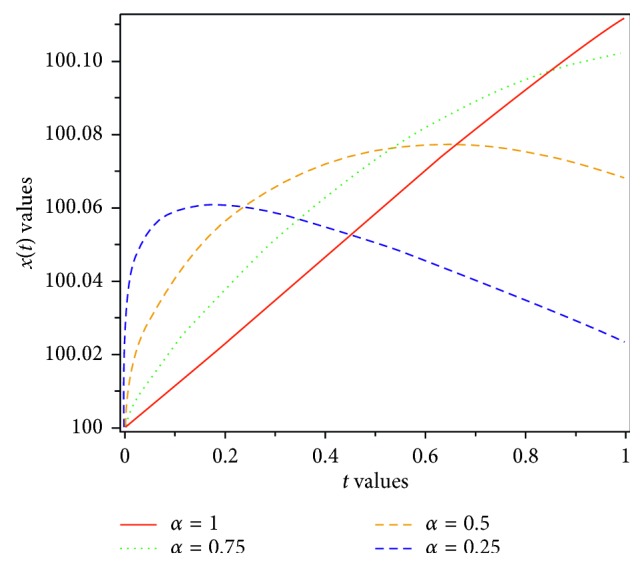
Dynamics of uninfected CD4^+^ T-cells for various values of *α*.

**Figure 2:**
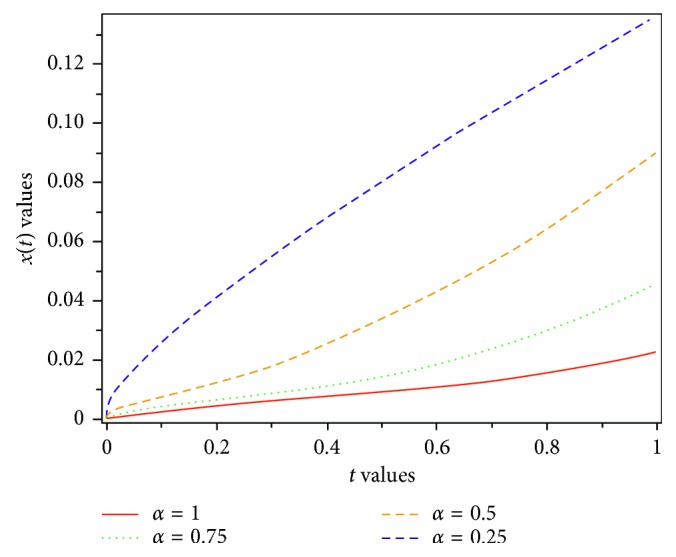
Dynamics of infected CD4^+^ T-cells for various values of *α*.

**Figure 3:**
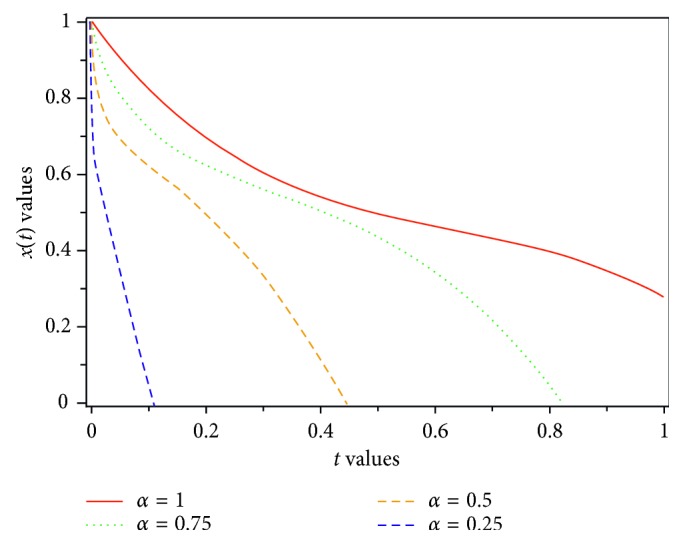
Dynamics of density of virions in plasma for various values of *α*.

Figures 1–3 show the results for different values of *α*, and the results can be compared.

## 5. Convergence Analysis of the Method

In this section, convergence of the proposed method, using the idea presented in [36], is studied.

Consider the following functional equation:

(22) $$\begin{array}{c} F\left(v\left(t\right)\right)=g\left(t\right), \end{array}$$

where *F* is a functional operator and can be decomposed as *F*=*D*^*α*^+*R*+*N* and *g* is a known function.

*D* ^*α*^ is a Caputo fractional derivative operator, *R* is a linear operator, and *N* is a nonlinear analytic operator, respectively. So equation (22) can be written as follows:

(23) $$\begin{array}{c} D^{\alpha }\left(v\left(t\right)\right)=g\left(t\right)-R\left(v\left(t\right)\right)-N\left(v\left(t\right)\right). \end{array}$$

The goal is to find a function *v*(*t*) satisfying equation (22). By applying the Laplace transform on both sides of equation (23) reads

(24) $$\begin{array}{c} ℒ\left\{D^{\alpha }\left(v\left(t\right)\right)\right\}=ℒ\left\{g\left(t\right)-R\left(v\left(t\right)\right)-N\left(v\left(t\right)\right)\right\}. \end{array}$$

By using definition (2.3), equation (23) can be written as follows:

(25) $$\begin{array}{c} ℒ\left\{v\left(t\right)\right\}=\frac{v\left(0\right)}{s}+\frac{ℒ\left\{g\left(t\right)\right\}}{s^{\alpha }}-\frac{ℒ\left\{R\left(v\left(t\right)\right)\right\}}{s^{\alpha }}-\frac{ℒ\left\{N\left(v\left(t\right)\right)\right\}}{s^{\alpha }}, \end{array}$$

by considering *v*(0)=*v*_0_ and using inverse of Laplace transform on both sides of equation (25) results in

(26) $$\begin{array}{c} {ℒ}^{-1}\left\{ℒ\left\{v\left(t\right)\right\}\right\}={ℒ}^{-1}\left\{\frac{v\left(0\right)}{s}+\frac{ℒ\left\{g\left(t\right)\right\}}{s^{\alpha }}-\frac{ℒ\left\{R\left(v\left(t\right)\right)\right\}}{s^{\alpha }}-\frac{ℒ\left\{N\left(v\left(t\right)\right)\right\}}{s^{\alpha }}\right\}, \end{array}$$

which implies that

(27) $$\begin{array}{c} v\left(t\right)=v_{0}+{ℒ}^{-1}\left\{\frac{ℒ\left\{g\left(t\right)\right\}}{s^{\alpha }}\right\}-{ℒ}^{-1}\left\{\frac{ℒ\left\{R\left(v\left(t\right)\right)\right\}}{s^{\alpha }}\right\}-{ℒ}^{-1}\left\{\frac{ℒ\left\{N\left(v\left(t\right)\right)\right\}}{s^{\alpha }}\right\}. \end{array}$$

By implementing ADM and assuming the solution *v*(*t*) as an infinite series say, *v*(*t*)=∑_*n*=0_^*∞*^*v*_*n*_(*t*), and writing the nonlinear term based on Adomian polynomials such as

(28) $$\begin{array}{c} N\left(v\left(t\right)\right)=\overset{\infty }{\underset{n=0}{\sum }}p_{n}\left(v_{0}\left(t\right),v_{1}\left(t\right),\ldots ,v_{n}\left(t\right)\right), \end{array}$$

where

(29) $$\begin{array}{c} p_{n}\left(v_{0}\left(t\right),v_{1}\left(t\right),\ldots ,v_{n}\left(t\right)\right)=\frac{1}{\Gamma \left(n+1\right)}\frac{d^{n}}{d\lambda^{n}}\left\{N_{\lambda }\right.{\left.\left(\overset{n}{\underset{i=0}{\sum }}v_{i}\left(t\right)\lambda^{i}\right)\right\}}_{\lambda =0}. \end{array}$$

Equation (27) can be written as the following form:

(30) $$\begin{array}{c} \overset{\infty }{\underset{n=0}{\sum }}v_{n}\left(t\right)=v_{0}+{ℒ}^{-1}\left\{\frac{ℒ\left\{g\left(t\right)\right\}}{s^{\alpha }}\right\}-{ℒ}^{-1}\left\{\frac{ℒ\left\{R\left(\sum_{n=0}^{\infty }v_{n}\left(t\right)\right)\right\}}{s^{\alpha }}\right\}-{ℒ}^{-1}\left\{\frac{ℒ\left\{\sum_{n=0}^{\infty }p_{n}\left(v_{0}\left(t\right),v_{1}\left(t\right),\ldots ,v_{n}\left(t\right)\right)\right\}}{s^{\alpha }}\right\}. \end{array}$$

So, we have

(31) $$\begin{array}{c} \overset{\infty }{\underset{n=0}{\sum }}v_{n}\left(t\right)=v_{0}+{ℒ}^{-1}\left\{\frac{ℒ\left\{g\left(t\right)\right\}}{s^{\alpha }}\right\}-\overset{\infty }{\underset{n=0}{\sum }}{ℒ}^{-1}\left\{\frac{ℒ\left\{R\left(v_{n}\left(t\right)\right)\right\}}{s^{\alpha }}\right\}-\overset{\infty }{\underset{n=0}{\sum }}{ℒ}^{-1}\left\{\frac{ℒ\left\{p_{n}\left(v_{0}\left(t\right),v_{1}\left(t\right),\ldots ,v_{n}\left(t\right)\right)\right\}}{s^{\alpha }}\right\}. \end{array}$$

From which we can define

(32) $$\begin{array}{c} \left\{\begin{array}{c} v_{0}\left(t\right)=v_{0},\\ v_{1}\left(t\right)={ℒ}^{-1}\left\{\frac{ℒ\left\{g\left(t\right)\right\}}{s^{\alpha }}\right\}-{ℒ}^{-1}\left\{\frac{ℒ\left\{R\left(v_{0}\left(t\right)\right)\right\}}{s^{\alpha }}\right\}-{ℒ}^{-1}\left\{\frac{ℒ\left\{p_{0}\left(v_{0}\left(t\right)\right)\right\}}{s^{\alpha }}\right\},\\ v_{2}\left(t\right)=-{ℒ}^{-1}\left\{\frac{ℒ\left\{R\left(v_{1}\left(t\right)\right)\right\}}{s^{\alpha }}\right\}-{ℒ}^{-1}\left\{\frac{ℒ\left\{p_{1}\left(v_{0}\left(t\right),v_{1}\left(t\right)\right)\right\}}{s^{\alpha }}\right\},\\ \vdots \\ v_{n+1}\left(t\right)=-{ℒ}^{-1}\left\{\frac{ℒ\left\{R\left(v_{n}\left(t\right)\right)\right\}}{s^{\alpha }}\right\}-{ℒ}^{-1}\left\{\frac{ℒ\left\{p_{n}\left(v_{0}\left(t\right),v_{1}\left(t\right),\ldots ,v_{n}\left(t\right)\right)\right\}}{s^{\alpha }}\right\}. \end{array}\right. \end{array}$$

**Theorem 1 .**

LADM for equation (21) with the solution (19) is equivalent to

(33) $$\begin{array}{c} s_{n}\left(t\right)=v_{0}\left(t\right)+v_{1}\left(t\right)+\ldots +v_{n}\left(t\right),\\ s_{0}\left(t\right)=v_{0}\left(t\right). \end{array}$$

By using the following iterative scheme:

(34) $$\begin{array}{c} s_{n+1}\left(t\right)=v_{0}+{ℒ}^{-1}\left\{\frac{ℒ\left\{g\left(t\right)\right\}}{s^{\alpha }}\right\}-{ℒ}^{-1}\left\{\frac{ℒ\left\{R\left(s_{n}\left(t\right)\right)\right\}}{s^{\alpha }}\right\}-{ℒ}^{-1}\left\{\frac{ℒ\left\{N\left(s_{n}\left(t\right)\right)\right\}}{s^{\alpha }}\right\}, \end{array}$$

where

(35) $$\begin{array}{c} N\left(\overset{n}{\underset{i=0}{\sum }}v_{i}\left(t\right)\right)=\overset{n}{\underset{i=0}{\sum }}p_{i}\left(v_{0}\left(t\right),v_{1}\left(t\right),\ldots ,v_{i}\left(t\right)\right),\;n=0,1,2,\ldots . \end{array}$$

**Proof**

For *n*=0, from equation (33):

(36) $$\begin{array}{c} s_{1}\left(t\right)=v_{0}+{ℒ}^{-1}\left\{\frac{ℒ\left\{g\left(t\right)\right\}}{s^{\alpha }}\right\}-{ℒ}^{-1}\left\{\frac{ℒ\left\{R\left(s_{0}\left(t\right)\right)\right\}}{s^{\alpha }}\right\}-{ℒ}^{-1}\left\{\frac{ℒ\left\{N\left(s_{0}\left(t\right)\right)\right\}}{s^{\alpha }}\right\}\\ =v_{0}+{ℒ}^{-1}\left\{\frac{ℒ\left\{g\left(t\right)\right\}}{s^{\alpha }}\right\}-{ℒ}^{-1}\left\{\frac{ℒ\left\{R\left(v_{0}\left(t\right)\right)\right\}}{s^{\alpha }}\right\}-{ℒ}^{-1}\left\{\frac{ℒ\left\{p_{0}\left(v_{0}\left(t\right)\right)\right\}}{s^{\alpha }}\right\}. \end{array}$$

Then, by assumption of equation (33), we have

(37) $$\begin{array}{c} v_{1}\left(t\right)={ℒ}^{-1}\left\{\frac{ℒ\left\{g\left(t\right)\right\}}{s^{\alpha }}\right\}-{ℒ}^{-1}\left\{\frac{ℒ\left\{R\left(v_{0}\left(t\right)\right)\right\}}{s^{\alpha }}\right\}-{ℒ}^{-1}\left\{\frac{ℒ\left\{p_{0}\left(v_{0}\left(t\right)\right)\right\}}{s^{\alpha }}\right\}, \end{array}$$

for *n*=1:

(38) $$\begin{array}{c} s_{2}\left(t\right)=v_{0}+{ℒ}^{-1}\left\{\frac{ℒ\left\{g\left(t\right)\right\}}{s^{\alpha }}\right\}-{ℒ}^{-1}\left\{\frac{ℒ\left\{R\left(s_{1}\left(t\right)\right)\right\}}{s^{\alpha }}\right\}-{ℒ}^{-1}\left\{\frac{ℒ\left\{N\left(s_{1}\left(t\right)\right)\right\}}{s^{\alpha }}\right\}\\ =v_{0}+{ℒ}^{-1}\left\{\frac{ℒ\left\{g\left(t\right)\right\}}{s^{\alpha }}\right\}-{ℒ}^{-1}\left\{\frac{ℒ\left\{R\left(v_{0}\left(t\right)+v_{1}\left(t\right)\right)\right\}}{s^{\alpha }}\right\}-{ℒ}^{-1}\left\{\frac{ℒ\left\{p_{0}\left(v_{0}\left(t\right)\right)+p_{1}\left(v_{0}\left(t\right),v_{1}\left(t\right)\right)\right\}}{s^{\alpha }}\right\}\\ =v_{0}\left(t\right)+v_{1}\left(t\right)-{ℒ}^{-1}\left\{\frac{ℒ\left\{R\left(v_{1}\left(t\right)\right)\right\}}{s^{\alpha }}\right\}-{ℒ}^{-1}\left\{\frac{ℒ\left\{p_{1}\left(v_{0}\left(t\right),v_{1}\left(t\right)\right)\right\}}{s^{\alpha }}\right\}. \end{array}$$

We know that *s*_2_(*t*)=*v*_0_(*t*)+*v*_1_(*t*)+*v*_2_(*t*), so we obtain

(39) $$\begin{array}{c} v_{2}\left(t\right)=-{ℒ}^{-1}\left\{\frac{ℒ\left\{R\left(v_{1}\left(t\right)\right)\right\}}{s^{\alpha }}\right\}-{ℒ}^{-1}\left\{\frac{ℒ\left\{p_{1}\left(v_{0}\left(t\right),v_{1}\left(t\right)\right)\right\}}{s^{\alpha }}\right\}. \end{array}$$

By strong induction, let us have

(40) $$\begin{array}{c} v_{k+1}\left(t\right)=-{ℒ}^{-1}\left\{\frac{ℒ\left\{R\left(v_{k}\left(t\right)\right)\right\}}{s^{\alpha }}\right\}-{ℒ}^{-1}\left\{\frac{ℒ\left\{p_{k}\left(v_{0}\left(t\right),v_{1}\left(t\right),\ldots ,v_{k}\left(t\right)\right)\right\}}{s^{\alpha }}\right\},\;k=1,2,\ldots ,n-1, \end{array}$$

and prove the following for *k*=*n*,

(41) $$\begin{array}{c} s_{n+1}\left(t\right)=v_{0}+{ℒ}^{-1}\left\{\frac{ℒ\left\{g\left(t\right)\right\}}{s^{\alpha }}\right\}-{ℒ}^{-1}\left\{\frac{ℒ\left\{R\left(s_{n}\left(t\right)\right)\right\}}{s^{\alpha }}\right\}-{ℒ}^{-1}\left\{\frac{ℒ\left\{N\left(s_{n}\left(t\right)\right)\right\}}{s^{\alpha }}\right\}\\ =v_{0}+{ℒ}^{-1}\left\{\frac{ℒ\left\{g\left(t\right)\right\}}{s^{\alpha }}\right\}-{ℒ}^{-1}\left\{\frac{ℒ\left\{R\left(\sum_{k=0}^{n}v_{k}\left(t\right)\right)\right\}}{s^{\alpha }}\right\}-{ℒ}^{-1}\left\{\frac{ℒ\left\{\sum_{k=0}^{n}p_{k}\left(v_{0}\left(t\right),v_{1}\left(t\right),\ldots ,v_{k}\left(t\right)\right)\right\}}{s^{\alpha }}\right\}\\ =v_{0}+{ℒ}^{-1}\left\{\frac{ℒ\left\{g\left(t\right)\right\}}{s^{\alpha }}\right\}-\overset{n}{\underset{k=0}{\sum }}{ℒ}^{-1}\left\{\frac{ℒ\left\{R\left(v_{k}\left(t\right)\right)\right\}}{s^{\alpha }}\right\}-\overset{n}{\underset{k=0}{\sum }}{ℒ}^{-1}\left\{\frac{ℒ\left\{p_{k}\left(v_{0}\left(t\right),v_{1}\left(t\right),\ldots ,v_{k}\left(t\right)\right)\right\}}{s^{\alpha }}\right\}\\ =v_{0}\left(t\right)+v_{1}\left(t\right)+\ldots v_{n}\left(t\right)-{ℒ}^{-1}\left\{\frac{ℒ\left\{R\left(v_{n}\left(t\right)\right)\right\}}{s^{\alpha }}\right\}-{ℒ}^{-1}\left\{\frac{ℒ\left\{p_{n}\left(v_{0}\left(t\right),v_{1}\left(t\right),\ldots ,v_{n}\left(t\right)\right)\right\}}{s^{\alpha }}\right\}. \end{array}$$

Then, from equation (33), we derive

(42) $$\begin{array}{c} v_{n+1}\left(t\right)=-{ℒ}^{-1}\left\{\frac{ℒ\left\{R\left(v_{n}\left(t\right)\right)\right\}}{s^{\alpha }}\right\}-{ℒ}^{-1}\left\{\frac{ℒ\left\{p_{n}\left(v_{0}\left(t\right),v_{1}\left(t\right),\ldots ,v_{n}\left(t\right)\right)\right\}}{s^{\alpha }}\right\}. \end{array}$$

This entails the statement is true and the theorem is proved.

**Theorem 2 .**

Let *X* be a Banach space.

- ∑_*i*=0_^*∞*^*v*_*i*_(*t*) resulted from equation (31), convergence to *s*  ∈  *X*, if ∃  *c* ∈ [0,1), s.t (∀*n*  ∈ℕ⟹‖*v*_*n*+1_‖ ≤ *c*  ‖*v*_*n*_‖),
- *s*(*t*)=∑_*i*=0_^*∞*^*v*_*i*_(*t*) satisfies in

(43) $$\begin{array}{c} s\left(t\right)=v_{0}+{ℒ}^{-1}\left\{\frac{ℒ\left\{g\left(t\right)\right\}}{s^{\alpha }}\right\}-{ℒ}^{-1}\left\{\frac{ℒ\left\{R\left(s\left(t\right)\right)\right\}}{s^{\alpha }}\right\}-{ℒ}^{-1}\left\{\frac{ℒ\left\{N\left(s\left(t\right)\right)\right\}}{s^{\alpha }}\right\}. \end{array}$$

**Proof**

(44) $$\begin{array}{c} \left\|s_{n+1}-s_{n}\right\|=\left\|v_{n+1}\right\|\leq c\left\|v_{n}\right\|\leq c^{2}\left\|v_{n-1}\right\|\leq \ldots \leq c^{n+1}\left\|v_{0}\right\|. \end{array}$$

*∀n*, *m*  ∈ℕ, *n* ≥ *m*, we have

(45) $$\begin{array}{c} \left\|s_{n}-s_{m}\right\|=\left\|\left(s_{n}-s_{n-1}\right)+\left(s_{n-1}-s_{n-2}\right)+\ldots +\left(s_{m+1}-s_{m}\right)\right\|\\ \leq \left\|s_{n}-s_{n-1}\right\|+\left\|s_{n-1}-s_{n-2}\right\|+\ldots +\left\|s_{m+1}-s_{m}\right\|\\ \leq c^{n}\left\|v_{0}\right\|+c^{n-1}\left\|v_{0}\right\|+\ldots +c^{m+1}\left\|v_{0}\right\|\leq \left(c^{n}+c^{n-1}+\ldots +c^{m+1}\right)\left\|v_{0}\right\|\\ \leq c^{m+1}\left(1+c+c^{2}+\ldots +c^{n}+\ldots \right)\leq \frac{c^{m+1}}{1-c}\left\|v_{0}\right\|. \end{array}$$

This means that $\underset{n,m⟶\infty }{\lim}\left\|s_{n}-s_{m}\right\|=0$, therefore, {*s*_*n*_} is a Cauchy sequence in the Banach space of *X* and is convergent. So, $\exists \;\;s\;\;\epsilon \;\;X,\text{s}.\text{t }\underset{n⟶\infty }{\lim}s_{n}=s$.

From equation (33), we derive

(46) $$\begin{array}{c} \underset{n⟶\infty }{\lim\;s_{n}\left(t\right)}=v_{0}+{ℒ}^{-1}\left\{\frac{ℒ\left\{g\left(t\right)\right\}}{s^{\alpha }}\right\}-{ℒ}^{-1}\left\{\frac{ℒ\left\{\underset{n⟶\infty }{\lim}R\left(s_{n}\left(t\right)\right)\right\}}{s^{\alpha }}\right\}-{ℒ}^{-1}\left\{\frac{ℒ\left\{\underset{n⟶\infty }{\lim}N\left(s_{n}\left(t\right)\right)\right\}}{s^{\alpha }}\right\}\\ =v_{0}+{ℒ}^{-1}\left\{\frac{ℒ\left\{g\left(t\right)\right\}}{s^{\alpha }}\right\}-{ℒ}^{-1}\left\{\frac{ℒ\left\{\underset{n⟶\infty }{\lim}R\left(\sum_{k=0}^{n}v_{k}\left(t\right)\right)\right\}}{s^{\alpha }}\right\}-{ℒ}^{-1}\left\{\frac{ℒ\left\{\underset{n⟶\infty }{\lim}N\left(\sum_{k=0}^{n}v_{k}\left(t\right)\right)\right\}}{s^{\alpha }}\right\}\\ =v_{0}+{ℒ}^{-1}\left\{\frac{ℒ\left\{g\left(t\right)\right\}}{s^{\alpha }}\right\}-{ℒ}^{-1}\left\{\frac{ℒ\left\{\underset{n⟶\infty }{\lim}\sum_{k=0}^{n}R\left(v_{k}\left(t\right)\right)\right\}}{s^{\alpha }}\right\}-{ℒ}^{-1}\left\{\frac{ℒ\left\{\underset{n⟶\infty }{\lim}\sum_{k=0}^{n}p_{k}\left(v_{0}\left(t\right),v_{1}\left(t\right),\ldots ,v_{k}\left(t\right)\right)\right\}}{s^{\alpha }}\right\}\\ =v_{0}+{ℒ}^{-1}\left\{\frac{ℒ\left\{g\left(t\right)\right\}}{s^{\alpha }}\right\}-{ℒ}^{-1}\left\{\frac{ℒ\left\{\sum_{k=0}^{\infty }R\left(v_{k}\left(t\right)\right)\right\}}{s^{\alpha }}\right\}-{ℒ}^{-1}\left\{\frac{ℒ\left\{\sum_{k=0}^{\infty }p_{k}\left(v_{0}\left(t\right),v_{1}\left(t\right),\ldots ,v_{k}\left(t\right)\right)\right\}}{s^{\alpha }}\right\}, \end{array}$$

From equation (35), we have

(47) $$\begin{array}{c} N\left(\overset{\infty }{\underset{i=0}{\sum }}v_{i}\left(t\right)\right)=\overset{\infty }{\underset{i=0}{\sum }}p_{i}\left(v_{0}\left(t\right),v_{1}\left(t\right),\ldots ,v_{i}\left(t\right)\right),\;n=0,1,2.\ldots . \end{array}$$

So,

(48) $$\begin{array}{c} s\left(t\right)=v_{0}+{ℒ}^{-1}\left\{\frac{ℒ\left\{g\left(t\right)\right\}}{s^{\alpha }}\right\}-{ℒ}^{-1}\left\{\frac{ℒ\left\{R\left(\sum_{k=0}^{\infty }v_{k}\left(t\right)\right)\right\}}{s^{\alpha }}\right\}-{ℒ}^{-1}\left\{\frac{ℒ\left\{N\left(\sum_{k=0}^{\infty }v_{k}\left(t\right)\right)\right\}}{s^{\alpha }}\right\}\\ =v_{0}+{ℒ}^{-1}\left\{\frac{ℒ\left\{g\left(t\right)\right\}}{s^{\alpha }}\right\}-{ℒ}^{-1}\left\{\frac{ℒ\left\{R\left(s\left(t\right)\right)\right\}}{s^{\alpha }}\right\}-{ℒ}^{-1}\left\{\frac{ℒ\left\{N\left(s\left(t\right)\right)\right\}}{s^{\alpha }}\right\}. \end{array}$$

**Lemma 2 .**

Equation (43) is equivalent to equation (22).

**Proof**

By using Laplace transform on both sides of equation (43) reads to

(49) $$\begin{array}{c} ℒ\left(s\left(t\right)\right)=ℒ\left(v_{0}\right)+\frac{ℒ\left\{g\left(t\right)\right\}}{s^{\alpha }}-\frac{ℒ\left\{R\left(s\left(t\right)\right)\right\}}{s^{\alpha }}-\frac{ℒ\left\{N\left(s\left(t\right)\right)\right\}}{s^{\alpha }}\\ =\frac{v\left(0\right)}{s}+\frac{ℒ\left\{g\left(t\right)\right\}}{s^{\alpha }}-\frac{ℒ\left\{R\left(s\left(t\right)\right)\right\}}{s^{\alpha }}-\frac{ℒ\left\{N\left(s\left(t\right)\right)\right\}}{s^{\alpha }}\\ =\frac{s^{\alpha -1}v\left(0\right)}{s^{\alpha }}+\frac{ℒ\left\{g\left(t\right)\right\}}{s^{\alpha }}-\frac{ℒ\left\{R\left(s\left(t\right)\right)\right\}}{s^{\alpha }}-\frac{ℒ\left\{N\left(s\left(t\right)\right)\right\}}{s^{\alpha }}, \end{array}$$

so we can write

(50) $$\begin{array}{c} s^{\alpha }ℒ\left(s\left(t\right)\right)-s^{\alpha -1}v\left(0\right)=ℒ\left\{g\left(t\right)\right\}-ℒ\left\{R\left(s\left(t\right)\right)\right\}-ℒ\left\{N\left(s\left(t\right)\right)\right\}. \end{array}$$

In virtue of definition 2.3 and linearity of the Laplace transform, equation (50) can be written as the follows:

(51) $$\begin{array}{c} ℒ\left\{D^{\alpha }s\left(t\right)\right\}=ℒ\left\{g\left(t\right)-R\left(s\left(t\right)\right)-N\left(s\left(t\right)\right)\right\}. \end{array}$$

By applying the inverse of Laplace transform on both sides of equation (51), we derive

(52) $$\begin{array}{c} D^{\alpha }s\left(t\right)=g\left(t\right)-R\left(s\left(t\right)\right)-N\left(s\left(t\right)\right). \end{array}$$

Considering *v*(*t*)=*s*(*t*), one gets equation (22). So, the solution of equation (43) is the same as the solution of equation (22).

## 6. Conclusion

In this paper, a fractional-order model of HIV-1 with three components has been introduced. When *α*⟶1, then *D*^*α*^*x*(*t*)⟶*Dx*(*t*); therefore, the fractional-order of presented model reduces to traditional model. By applying Laplace transform and Adomian decomposition method, or LADM for short, which is a strong approach to compute numerical solution of fractional differential equations, we gain an approximate solution of the proposed model. The accuracy of the proposed approach has made it a reliable method. We have calculated four terms of the infinite series of *x*, *y*, and *z* as an approximate solution. The result of LADM has been compared with the results of some other methods such as GEM, HAM, RK4 [8], and HPM [31]. The results are presented in Tables 1–3. Figures 1–3, show that the uninfected CD4^+^ T-cells, *x*, infected CD4^+^ T-cells, *y*, and density of virions in plasma, *z*, depend on the various values of *α*, the various values of the parameters, and the time fractional derivative. A comparison of the approximate solutions shows that LADM can work more accurate than other methods. Convergence of the proposed method is studied. Because of the fact that obtaining the exact solution for system of fractional equation is difficult or impossible, we would like to suggest such an easy and reliable approach for further research, in the future.

## Figures and Tables

**Table 1:** Numerical results of *x*(*t*) (uninfected CD4^+^ T-cells).

*t*	LADM	GEM	HPM	HAM	RK4
0	100	100	100	100	100
0.2	100.023	100.023	100.023	100.023	100.023
0.4	100.046	100.047	100.047	100.047	100.047
0.6	100.070	100.071	100.071	100.071	100.071
0.8	100.092	100.097	100.097	100.096	100.097
1.0	100.112	100.122	100.123	100.122	100.122

**Table 2:** Numerical results of *y*(*t*) (infected CD4^+^ T-cells).

*t*	LADM	GEM	HPM	HAM	RK4
0	0	0	0	0	0
0.2	0.00436	0.00434	0.00434	0.00434	0.00434
0.4	0.00750	0.00715	0.00721	0.00714	0.00715
0.6	0.01074	0.00908	0.00934	0.00909	0.00908
0.8	0.01336	0.01049	0.01117	0.01063	0.01049
1.0	0.01866	0.01161	0.01276	0.01194	0.01161

**Table 3:** Numerical results of *z*(*t*) (density of virions in plasma).

*t*	LADM	GEM	HPM	HAM	RK4
0	1	1	1	1	1
0.2	0.69581	0.69030	0.69071	0.69059	0.69070
0.4	0.53851	0.51152	0.51208	0.51237	0.51190
0.6	0.46097	0.41069	0.41394	0.40994	0.41103
0.8	0.39607	0.35656	0.37749	0.35148	0.35684
1.0	0.27671	0.33053	0.42419	0.32869	0.33073

## Data Availability

Data used to support this study are available at DOI: https://doi.org/10.1038/sj.icb.7100056. These prior studies (and datasets) are cited at relevant places within the text as references [6, 8, 16].
